# P-1399. Impact of Positive Chlamydia and Gonorrhea Testing Among Male Military Trainees

**DOI:** 10.1093/ofid/ofae631.1574

**Published:** 2025-01-29

**Authors:** Rachel E Powers, Erin Winkler, Theresa Casey, Ga O Jung, Angela Osuna, Joseph Marcus

**Affiliations:** San Antonio Uniformed Services Health Education Consortium, San Antonio, Texas; BAMC, San Antonio, Texas; BAMC, San Antonio, Texas; 559th Medical Group, JBSA-Lackland, Texas; BAMC, San Antonio, Texas; Brooke Army Medical Center, San Antonio, TX

## Abstract

**Background:**

All females trainees entering the U.S. Air Force basic military training (BMT) are tested for chlamydia (CT) and gonorrhea (GC) and account for the vast majority of cases in this population. Recent studies have demonstrated that, when universally tested, BMT males had similar overall rates of CT and GC as compared to females, as most cases are asymptomatic. This study evaluates the incidence of CT and GC in male BMT trainees and follow-up.

**Figure:**
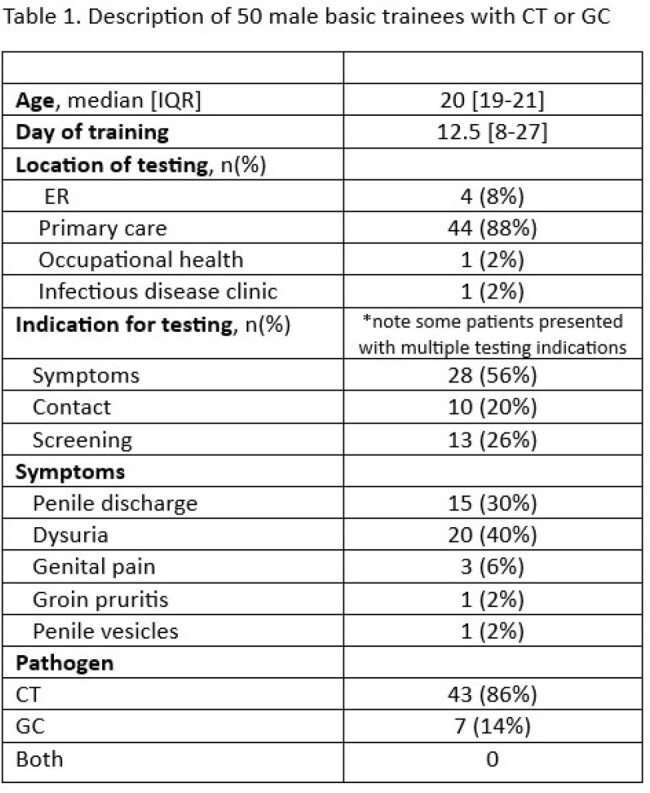

**Methods:**

Over 30,000 BMT recruits train at Joint Base San Antonio-Lackland each year. This study included all male trainees who tested positive for CT or GC during their 8-week training period between 2017-2023. Information was obtained regarding patient’s age, indication for testing, facility-type performing testing, date of test, follow-up testing, and occurrence of STIs within three years of diagnosis. Male trainees were then matched with female trainees based on age, date of diagnosis, and pathogen type to determine sex-based differences in follow-up.

**Figure:**
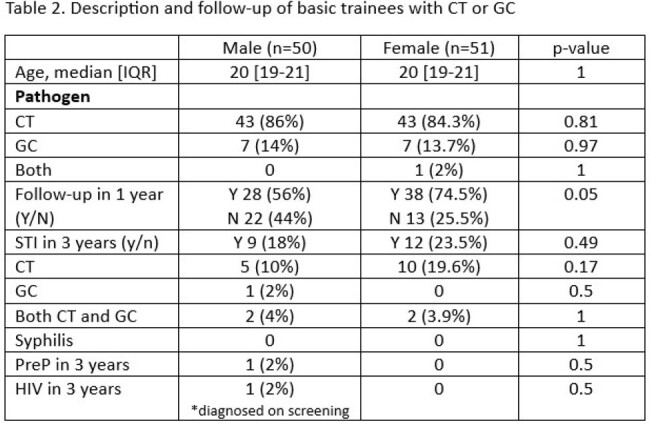

**Results:**

50 (0.03%) of the 182,726 males who attended BMT between 2017-2023 tested positive for CT or GC during their training. Most cases (88%) were detected in primary care and the median time in training until diagnosis was 12.5 [IQR: 8-27] days. (56%) of male trainees had symptoms on presentation for STI testing, with dysuria (40%) and penile discharge (35%) being the most common. When compared to matched-females, male trainees had significantly less follow-up testing within one year as compared to female trainees (56% vs. 75%, p=0.05). Despite the difference in follow-up testing, there was no difference in STIs diagnosed in the next three years (18% vs. 24%, p=0.5).

**Conclusion:**

Despite universal access to care, only slightly more than half of male BMT trainees with CT or GC received guideline-directed follow-up within one year. Additionally, approximately one in five men who tested positive for CT or GC during BMT developed a subsequent STI in the next three years, at rates similar to women who tested positive, despite less frequent testing. More work is needed to improve follow-up testing services for those who test positive for CT or GC.

**Disclosures:**

**All Authors**: No reported disclosures

